# Combining IC_50_ or *K*_*i*_ Values from Different Sources Is a Source of Significant Noise

**DOI:** 10.1021/acs.jcim.4c00049

**Published:** 2024-02-23

**Authors:** Gregory A. Landrum, Sereina Riniker

**Affiliations:** Department of Chemistry and Applied Biosciences, ETH Zurich, Vladimir-Prelog-Weg 2, 8093 Zurich, Switzerland

## Abstract

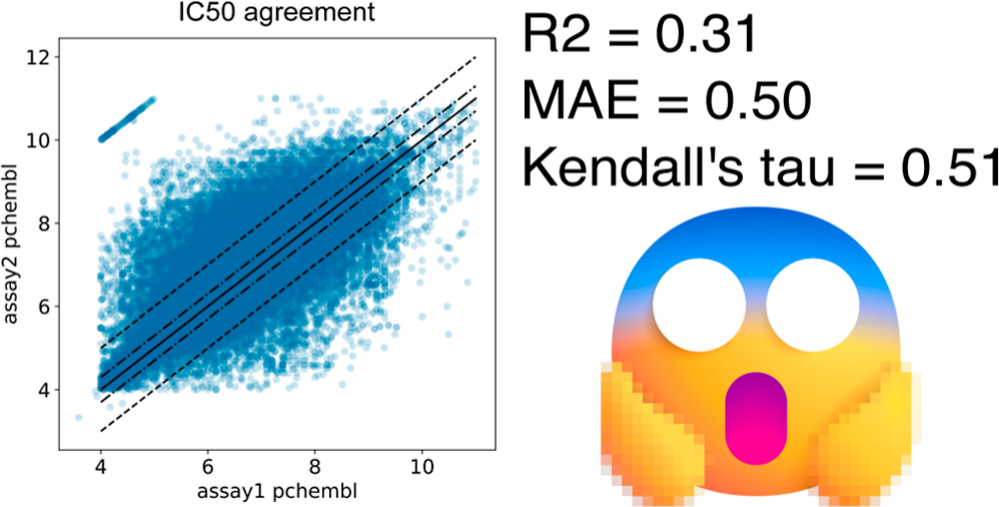

As part of the ongoing quest to find or construct large data sets for use in validating new machine learning (ML) approaches for bioactivity prediction, it has become distressingly common for researchers to combine literature IC_50_ data generated using different assays into a single data set. It is well-known that there are many situations where this is a scientifically risky thing to do, even when the assays are against exactly the same target, but the risks of assays being incompatible are even higher when pulling data from large collections of literature data like ChEMBL. Here, we estimate the amount of noise present in combined data sets using cases where measurements for the same compound are reported in multiple assays against the same target. This approach shows that IC_50_ assays selected using minimal curation settings have poor agreement with each other: almost 65% of the points differ by more than 0.3 log units, 27% differ by more than one log unit, and the correlation between the assays, as measured by Kendall’s τ, is only 0.51. Requiring that most of the assay metadata in ChEMBL matches (“maximal curation”) in order to combine two assays improves the situation (48% of the points differ by more than 0.3 log units, 13% by more than one log unit, and Kendall’s τ is 0.71) at the expense of having smaller data sets. Surprisingly, our analysis shows similar amounts of noise when combining data from different literature *K*_*i*_ assays. We suggest that good scientific practice requires careful curation when combining data sets from different assays and hope that our maximal curation strategy will help to improve the quality of the data that are being used to build and validate ML models for bioactivity prediction. To help achieve this, the code and ChEMBL queries that we used for the maximal curation approach are available as open-source software in our GitHub repository, https://github.com/rinikerlab/overlapping_assays.

## Introduction

**Figure 1:**
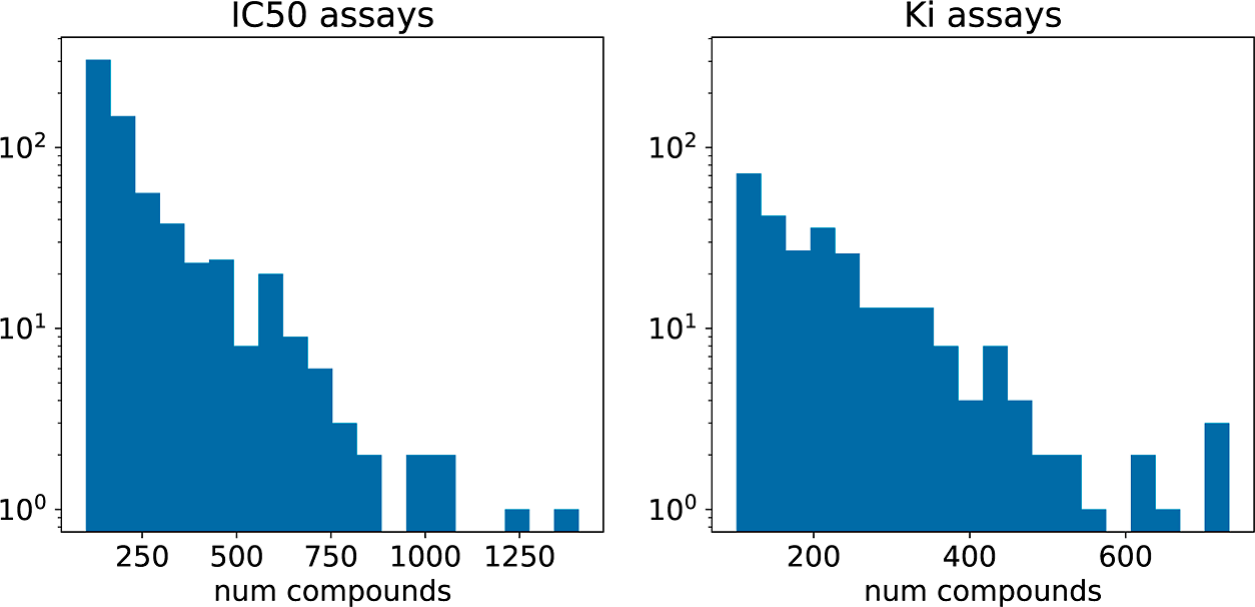
Histograms of the number of compounds per assay in ChEMBL32: IC_50_ assays (left) and *K*_*i*_ assays (right). Only measurements with a non-null *pchembl* value were included. Assays with 100 or less points are not included in these histograms.

Most artificial intelligence/machine learning (AI/ML) methods are very data hungry: they require a large amount of training data in order to build useful predictive models. Additionally, noise in the training data for the models sets an upper limit on the accuracy that can be expected. At the same time, there are not many large open data sets available that are applicable to computational drug discovery. Large, consistently measured data sets are typically only available inside companies and, due primarily to IP concerns, are difficult/impossible to publish in the open scientific literature. There are notable exceptions to this,^1,2^ but they are definitely rare. This has consequences for researchers who have access to only public data sources. For example, when extracting data from ChEMBL,^3,4^ the only way to be mostly certain that a data set was consistently measured is to only take data from a single assay. Unfortunately, more than 60,000 of the >85,000 IC_50_ assays in ChEMBL32 have data for less than 10 distinct compounds, only 650 assays have data for more than 100 distinct compounds, and there are only 54 assays with data for more than 500 distinct compounds (Figure 1). This dearth of large, consistent data sets has led to the common practice of combining results from different assays (measured against the same target) to create data sets for AI/ML applications.

### Compatibility Issues

Experimental data inevitably contains some noise; this is true even in the best case situation, where we are looking at data taken from the same assay measured in the same lab. The noise level rises when we compare experimental results from different laboratories due to small (or large) differences in assay protocols, reagents, etc. Variability is higher with some assay types than others, for example, Caco-2 permeability assays are well-known to have problems with interlab variability due to differences in the cells used in the assay as well as the impossibility of exactly reproducing experimental conditions when working with living systems.^5,6^ Looking beyond laboratory-to-laboratory variability of assays that are nominally the same, there are numerous reasons why literature results for different assays measured against the same “target” may not be comparable. These include the following:

1. Different assay conditions: these can include different buffers, experimental pH, temperature, and duration.
2. Substrate identity and concentration: these are particularly relevant for IC_50_ values from competition assays, where the identity and concentration of the substrate being competed with play an important role in determining the results. K_*i*_ measures the binding affinity of a ligand to an enzyme and so its values are, in principle, not sensitive to the identity or concentration of the substrate.
3. Different assay technologies: since typical biochemical assays do not directly measure ligand–protein binding, the idiosyncrasies of different assay technologies can lead to different results for the same ligand–protein pair.^7^
4. Mode of action for receptors: EC_50_ values can correspond to agonism, antagonism, inverse agonism, etc.

The situation is further complicated when working with databases like ChEMBL, which curate literature data sets:

1. Different targets: different variants of the same parent protein are assigned the same target ID in ChEMBL
2. Different assay organism or cell types: the target protein may be recombinantly expressed in different cell types (the target ID in ChEMBL is assigned based on the original source of the target), or the assays may be run using different cell types.
3. Any data source can contain human errors like transcription errors or reporting incorrect units. These may be present in the original publication—when the authors report the wrong units or include results from other publications with the wrong units—or introduced during the data extraction process.

All of these sources of variability in the measurements of activity values against the “same” target in different assays, incompatible assays, interlaboratory differences, experimental errors, etc., contribute noise to a combined data set that is intended to be used for bioactivity modeling. This noise inevitably decreases the quality and accuracy of models trained on the data.

In this work, we focus primarily on two of the largest classes of publicly available dose–response bioactivity data: IC_50_ and *K*_*i*_. IC_50_ measures the concentration of a compound required to inhibit a particular biological response, e.g., an enzymatic reaction or signaling by a receptor, by half (50%). *K*_*i*_, on the other hand, measures the equilibrium dissociation constant of a compound bound to a protein.^7^ The conventional wisdom is that it is generally not scientifically valid to combine values from different IC_50_ assays without knowledge of the assay conditions but that *K*_*i*_ values are more comparable across assays. Reference (8) provides a good explanation of the relationship between IC_50_ and *K*_*i*_.

### Assessing Assay Compatibility

The best way to determine whether the results from two different IC_50_ or *K*_*i*_ assays measured on the “same” target are compatible with each other is to read the original publications and directly assess whether all important parameters are the same. However, given the number of available IC_50_ assays for many targets (in ChEMBL32, human CDK2 has 343 assays, human BRD4 has 454 assays, and a common target like hERG has 2020 assays) this is not feasible at any sort of scale, so we need other compatibility metrics. One approach that lends itself to both automation and large-scale analysis is to identify pairs of assays in which the same compound (or multiple compounds) has been tested. Comparing the measured IC_50_ or *K*_*i*_ values for the compound(s) shared between the assays gives good sense as to whether or not the rest of the results can be compared. Results differing by less than an expected window for experimental error—for example ΔpIC_50_ < 0.3,^9−11^ approximately a factor of two—support the hypothesis that the assays are compatible.

In this work, we start by estimating the compatibility of the IC_50_ and *K*_*i*_ assays for the same target drawn from ChEMBL32. We then develop a curation methodology that takes advantage of the assay metadata available in ChEMBL to avoid combining results from assays that are clearly incompatible. The impact of this “max curation” scheme on data set quality and size is estimated and discussed.

## Methods

### Extracting Data from ChEMBL32

Data was extracted from a local copy of ChEMBL32^4^ running in a PostgreSQL database^12^ using standard SQL queries within the Jupyter computational notebook environment. The database was constructed directly, without modification, from the PostgreSQL dump provided by the ChEMBL team.^13^ All queries used can be found in the Jupyter notebooks in the project GitHub repository: https://github.com/rinikerlab/overlapping_assays.

### Quantifying Assay Compatibility

The compatibility between the two assays was measured by comparing *pchembl* values of overlapping compounds. In addition to plotting the values, a number of metrics were used to quantify the degree of compatibility between assay pairs:

- *R*^2^: the coefficient of determination provides a direct measure of how well the “duplicate” values in the two assays agree with each other. Values range from −1.0 to 1.0 with larger values corresponding to higher compatibility.
- Kendall τ: nonparametric measure of how equivalent the rankings of the measurements in the two assays are. Values range from −1.0 to 1.0 with larger values corresponding to higher compatibility.
- *f* > 0.3: fraction of the pairs where the difference is above the estimated experimental error. Smaller values correspond to higher compatibility.
- *f* > 1.0: fraction of the pairs where the difference is more than one log unit. This is an arbitrary limit for a truly meaningful activity difference. Smaller values correspond to higher compatibility.
- κ_bin_: Cohen’s κ calculated between the assays after binning their results into active and inactive using *bin* as the activity threshold. Values range from −1.0 to 1.0 with larger values corresponding to higher compatibility.
- MCC_bin_: Matthew’s correlation coefficient calculated between the assays after binning their results into active and inactive using *bin* as the activity threshold. Values range from −1.0 to 1.0 with larger values corresponding to higher compatibility.

All metrics were calculated using either scikit-learn^14^ version 1.2.2 or SciPy^15^ version 1.10.1.

### Curation Approaches

Given the obvious scientific problems and amount of noise introduced by combining all IC_50_ data (see the Results and Discussion section below), we explored a number of different strategies for more carefully curating the combined IC_50_ data sets based purely on the information available in the ChEMBL database.

The curation operations we applied were as follows:

- Activity curation: Pairs of measurements where the *pchembl* values in the two assays were either exactly the same or differed by 3.0 were removed. Given the very low probability of two separate experiments producing exactly the same results, the exact matches are most likely cases where values from a previous paper are copied into a new one; this was discussed in the earlier work by Kramer et al.^10^ and spot-checked with a number of assay pairs here. The pairs differing by exactly three log units correspond to the same copy action with the twist that a unit error was made in either one of the publications or during the ingestion into ChEMBL.
- Duplicate papers: Pairs of measurements where both assays were published in the same document were removed. Having two (or more) IC_50_ assays against the same target in the same paper usually only occurs when there is a difference between the two assays: either they have been run under different conditions or using different variants of the same protein (ChEMBL’s curation does not always distinguish between variants), etc.
- Remove mutants: because the ChEMBL target metadata does not provide information about variant proteins (still often called “mutants”), different variants of a target protein will share the same target ID as the wild type. However, the assay description field in ChEMBL will often contain some information about which variant was used. Before the release of ChEMBL22, this information was not captured systematically or using a controlled vocabulary. More recent versions of ChEMBL include the *variant_id* field in the assay metadata, so it is theoretically possible to detect similar variants for more recent assays. We adopt a conservative approach in this curation step and remove any assay that has the text “mutant”, “mutation”, or “variant” in its description or that has a variant ID specified.
- Assay type: one of the more important pieces of metadata that ChEMBL provides about assays is the assay type. This can take on values like “Binding”, “Functional”, “Physicochemical”, etc. This curation step removes pairs of assays with different assay types.
- Assay metadata: this curation step removes pairs of assays where any of the following assay metadata fields do not match: *assay_type, assay_organism*, *assay_category, assay_tax_id*, *assay_strain, assay_tissue*, *assay_cell_type*, *assay_subcellular_fraction*, and *bao_format.* This list covers almost all of the assay metadata fields available in ChEMBL32 and not already mentioned above.
- Sources other than documents: this curation step removes any assay that is from a source that does not have an associated document date. The goal here is to only include data sets from the medicinal chemistry literature and patents, excluding screening data sets or other contributed data sets.
- Assay size: by default, any assays that include >100 compounds are removed. The goal of this step is to try and focus attention on the primary literature and ignore sources like review articles. Because the upper limit is a very heuristic threshold, we have also explored (and included the data from) an upper limit of 1000 compounds.
- Curation confidence: when this curation step is enabled, any assay that does not have a confidence score value of 9 (indicating that the assay is assigned to a direct single target) is removed.

The impacts of each of these steps individually on the number of IC_50_ assay- and compound-pairs from ChEMBL32 are shown in Table S1 in the Supporting Information.

### Applying Maximal Curation to Extract Data Sets

The main goal of this work is to identify curation settings for extracting reliable (i.e., less noisy) data from ChEMBL. Once we have identified the appropriate settings, the data sets themselves need to be extracted. This task is easy when doing minimum curation: we simply retrieve all of the IC_50_ (or *K*_*i*_) data sets for a given target and combine them into a single data set labeled with the target ID. When doing maximal curation, we are more restrictive about which assays are considered: once we have identified the assays to be considered for a target, we create a “conditions hash” for each one. This is the md5 hash of the available assay metadata: *assay_type*, *assay_organism*, *assay_category*, *assay_tax_id*, *assay_strain, assay_tissue*, *assay_cell_type*, *assay_subcellular_fraction*, *bao_format*, and *variant_id*. The combination of target ID and condition hash defines a set of assays that are equivalent as far as we can tell from the information available in ChEMBL32. The final step is to combine these assays and label them with the target ID and conditions hash.

- Only assays associated with documents are considered.
- Only assays with a curation confidence score of 9 are considered.
- Assays with the text “mutant”, “mutation”, or “variant” in their descriptions are removed unless they have a non-null *variant_id.*
- If a document contains multiple assays against the same target, only the one with results for the largest number of compounds is retained.

For both curation settings, only unqualified activity values with nM standard values, non-null *pchembl* values, and no *data_validity_comment* are used.

## Results and Discussion

### Noise Introduced by Combining Assays

**Figure 2:**
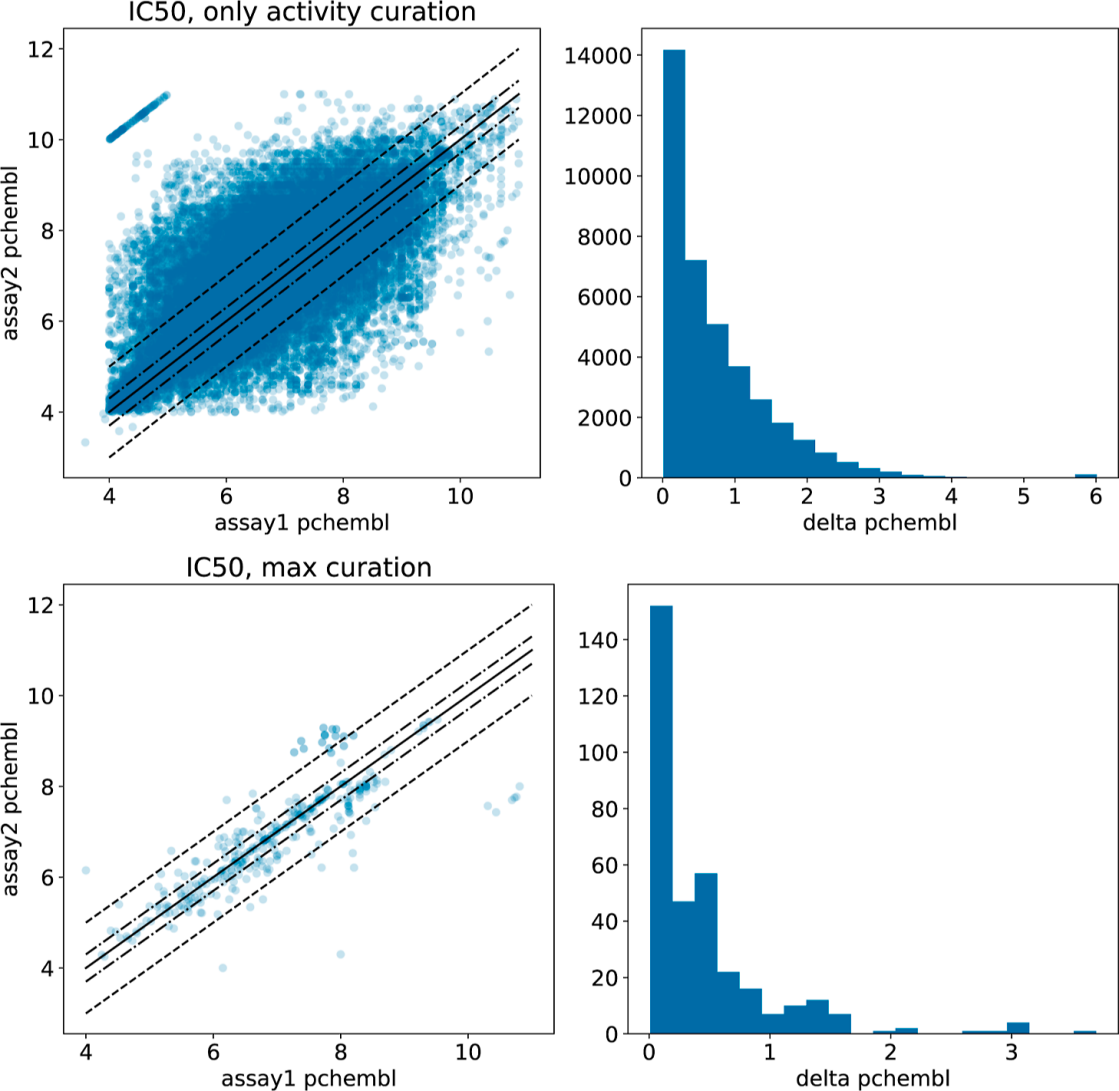
Agreement between duplicate measurements in IC_50_ assays on the same target with “only activity” curation (top) and maximal curation (bottom). (Left): correlation plot between *pchembl* values from the two assays. The solid black line corresponds to *x* = *y*, the dot-dashed lines mark a difference of 0.3, and the dashed line marks a difference of 1.0. (Right): histogram of Δpchembl, the differences in *pchembl* values.

We first looked at the variation in the data sets when IC_50_ assays are combined using “only activity” curation (top panels in Figure 2). The noise level in this case is very high: 64% of the Δpchembl values are greater than 0.3, and 27% are greater than 1.0. The analogous plot for the *K*_*i*_ data sets is shown in Figure S1 in the Supporting Information. The noise level for *K*_*i*_ is comparable: 67% of the Δpchembl values are greater than 0.3, and 30% are greater than 1.0. In Figure 2 and all similar plots in this study, the points are plotted such that the assay on the *x*-axis has a higher assay_id (this is the assay key in the SQL database, not the assay ChEMBL ID that is more familiar to users of the ChEMBL web interface) in ChEMBL32 than the assay on the *y*-axis. Given that assay_ids are assigned sequentially in the ChEMBL database, this means that the *x*-value of each point is most likely from a more recent publication than the *y*-value. We do not believe that this fact introduces any significant bias into our analysis.

The situation for IC_50_ improves markedly when using the maximal curation scheme, at the expense of discarding almost 99% of the data (bottom panels in Figure 2). τ increases from 0.51 to 0.71, and the MAE decreases from 0.50 to 0.27. Note that even with the maximal curation settings, 48% of the Δpchembl values differ by more than 0.3 log units, and 13% differ by more than 1.0.

**Figure 3:**
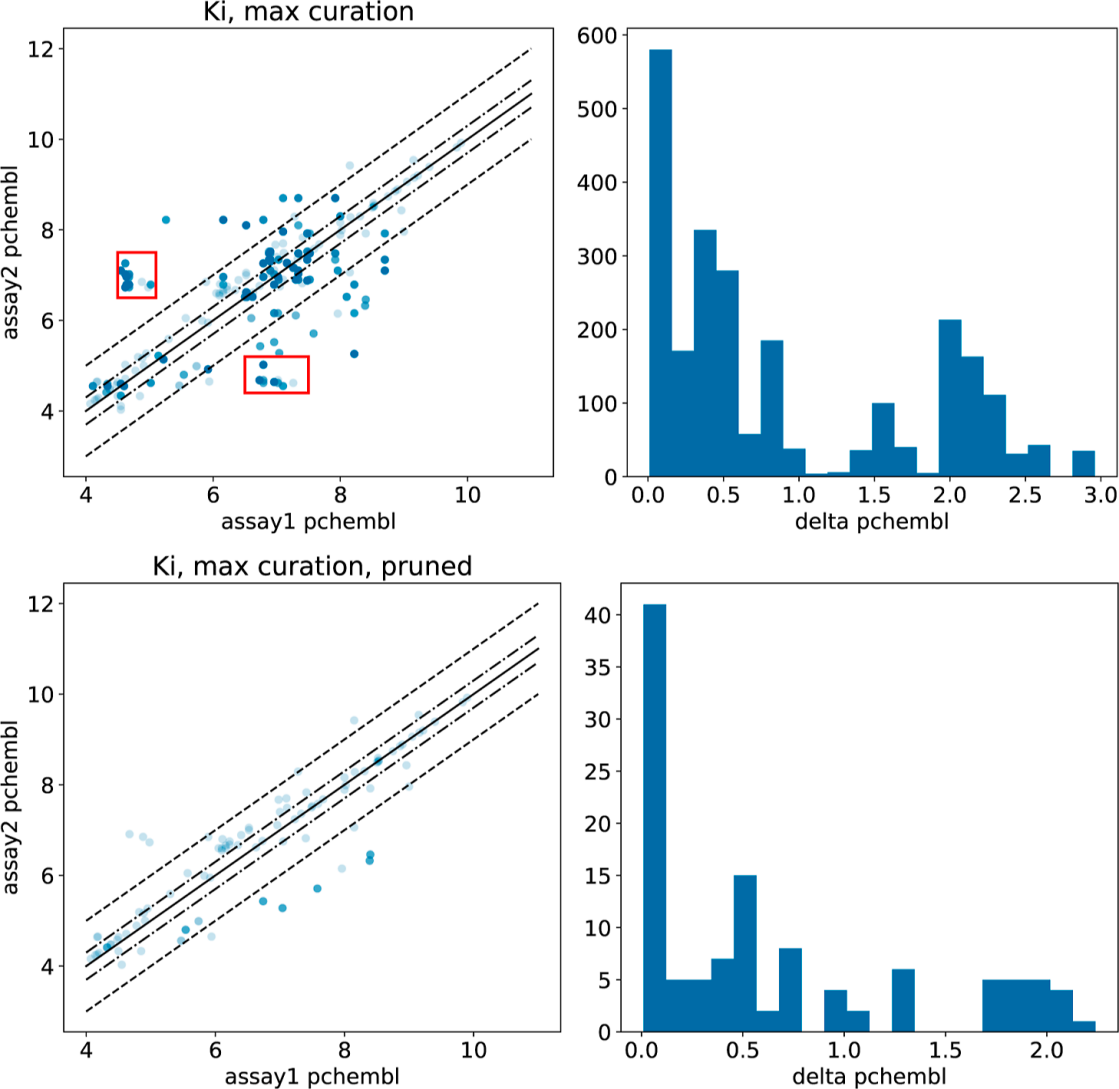
Agreement between duplicate measurements in *K*_*i*_ assays on the same target with maximal curation (top) and with 239 problematic assays (see text) removed (bottom). (Left): correlation plot between *pchembl* values from the two assays. The solid black line corresponds to *x* = *y*, the dot–dashed lines mark a difference of 0.3, and the dashed line marks a difference of 1.0. The regions outlined with red boxes are discussed in the text. (Right): histogram of Δpchembl, the differences in *pchembl* values.

The top panels of Figure 3 show a plot similar to Figure 2 for the *K*_*i*_ data sets with the maximal curation scheme. Here, we have only lost 70% of the data and have not improved the quality of the results over activity-only curation: 69% of the Δpchembl values are greater than 0.3, and 32% are greater than 1.0. Surprisingly, when it comes to the regression parameters presented in Table 1, the maximal curation results are actually worse than those from activity-only curation. What is happening here?

**Table 1:** Impact of Curation Level on Regression Quality Metrics^a^

readout	curation level	#assays	#Cmpds	*R*^2^	τ	MAE	*f* > 0.3	*f* > 1.0
IC_50_	only activity	1358	38,022	0.31	0.51	0.50	0.64	0.27
IC_50_	maximal	26	340	0.63	0.71	0.27	0.48	0.13
IC_50_ large	only activity	1599	50,385	0.32	0.51	0.51	0.65	0.28
IC_50_ large	maximal	44	742	0.60	0.61	0.30	0.51	0.15
*K*_*i*_	only activity	587	7734	0.13	0.43	0.52	0.67	0.30
*K*_*i*_	maximal	282	2434	–0.33	0.27	0.47	0.69	0.32
*K*_*i*_	maximal + pruning	9	115	0.65	0.67	0.45	0.58	0.25
*K*_*i*_ large	only activity	750	9650	0.21	0.46	0.46	0.64	0.27
*K*_*i*_ large	maximal	290	2574	–0.10	0.32	0.47	0.67	0.32
*K*_*i*_ large	maximal + pruning	17	255	0.68	0.71	0.12	0.38	0.21

a See the Methods section for a description of the metrics themselves. “Large” indicates when assays with up to 1000 compounds were included.

The top left panel of Figure 3 has two dense clusters of points that are highlighted in red boxes. These points arise from a set of 32 assays reporting *K*_*i*_ values for human carbonic anhydrase I (ChEMBL target ID CHEMBL261). These assays share a corresponding author and include a significant number of overlapping compounds, with results that are sometimes inconsistent. The original papers do not provide sufficient information about the sources of the data to understand the causes of this variability.^16,17^ Because this is almost certainly artificial variability and not just experimental noise, we removed all data from assays that have more than ten compounds in common with one of these assays (CHEMBL3782909^18^) from consideration and repeated the statistical analysis. The complete list of 239 assays removed from consideration is reported in the Supporting Information. The bottom panels of Figure 3 show the comparison with these assays removed. Most of the outliers are no longer present, and the agreement is significantly better (Table 1). Note that we were only able to be certain that there was a problem with these data by going back to the original publications. Resolving situations like this is a nontrivial curation exercise, which is difficult to automate. We mention it here as an illustration of the kinds of things that can go wrong even after doing maximal curation for “best case” experimental readouts, such as *K*_*i*_ data. Although we could reasonably expect *K*_*i*_ values to be at least somewhat comparable across laboratories, we were limited in this case by the quality of the data in the primary scientific literature.

The similarity in noise levels between the IC_50_ and *K*_*i*_ data sets, though still surprising, has been previously reported.^11^ In ref (11), and its predecessor study focusing on *K*_*i*_ data,^10^ an extensive amount of curation was carried out to identify pairs of points measured in different laboratories against the same target. The authors explicitly point out the noise introduced by blindly combining data from different IC_50_ assays. Note that limiting comparisons to assays performed in different laboratories automatically prevents the comparison of assays drawn from the same paper, one of the important pieces of our maximal curation procedure.

### Regression versus Classification

The previous results demonstrated the amount of noise that activity-only curation introduces to the IC_50_ values that would be used to build a regression model. What is the impact of this noise when we bin the activity data as we do when we build classification models? Table 2 shows κ and MCC values for three activity binning levels commonly used in the literature: *pchembl* = 5 (10 μM), *pchembl* = 6 (1 μM), and *pchembl* = 7 (100 nM). With the activity-only curation setting, the MCC values for all three thresholds are <0.6. Maximal curation improves the situation somewhat with MCC values ranging from 0.83 to 0.91. Similar improvements are observed for the *K*_*i*_ data when maximal curation is used together with pruning of the suspect assays.

**Table 2:** Impact of the Curation Level on Classification Quality Metrics^a^

readout	curation level	κ_5_	MCC_5_	κ_6_	MCC_6_	κ_7_	MCC_7_
IC_50_	only activity	0.50	0.50	0.56	0.57	0.55	0.56
IC_50_	max	0.83	0.83	0.87	0.87	0.91	0.91
IC_50_ large	only activity	0.50	0.51	0.56	0.56	0.51	0.52
IC_50_ large	max	0.73	0.73	0.84	0.84	0.78	0.78
*K*_*i*_	only activity	0.40	0.40	0.47	0.47	0.42	0.42
*K*_*i*_	max	0.15	0.15	0.27	0.27	0.20	0.20
*K*_*i*_	max + pruning	0.65	0.64	0.69	0.69	0.59	0.61
*K*_*i*_ large	only activity	0.48	0.48	0.52	0.52	0.46	0.46
*K*_*i*_ large	max	0.16	0.16	0.28	0.28	0.23	0.23
*K*_*i*_ large	max + pruning	0.66	0.66	0.73	0.73	0.64	0.64

a See the Methods section for a description of the metrics themselves. Three activity binning levels were considered: *pchembl* = 5 (10 μM), *pchembl* = 6 (1 μM), and *pchembl* = 7 (100 nM). “Large” indicates when assays with up to 1000 compounds were included.

The MCC and Cohen’s κ in Table 2 have very similar values because the confusion matrices are generally quite symmetric.^19^ This makes sense, given that the ordering of the assays by ChEMBL ID should not introduce any systematic differences in the two *pchembl* values.

### Impact of Curation on Data Set Size

**Figure 4:**
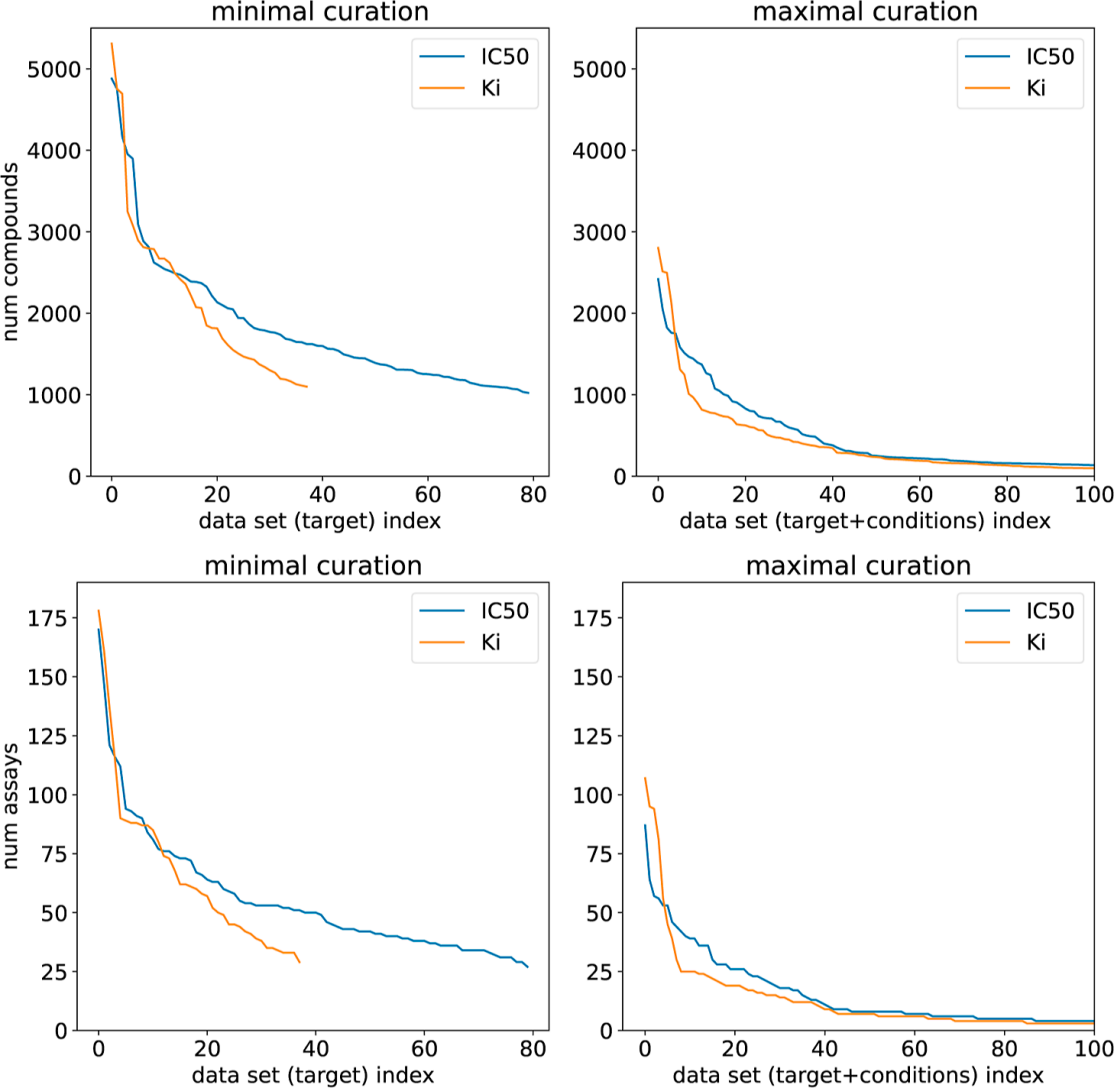
Number of compounds per combined data set (top) and number of assays per combined data set (bottom) for activity-only curation (left) and maximal curation (right). In each plot, the data sets are sorted by decreasing size. The right panel is truncated at 100 data sets to aid visibility.

As the maximal curation scheme seems to improve data quality (although it does not remove the noise in the data), we next investigated its impact on the size and composition of combined data sets from ChEMBL32. We started by using the activity-only curation settings to construct combined data sets for all targets that contained at least 20 assays and activity values for at least 1000 compounds. This yields 80 targets for IC_50_ and 38 targets for *K*_*i*_. The top panels of Figure 4 show the numbers of compounds in combined data sets using the activity-only and maximal curation settings, whereas the bottom panels show the number of assays combined into each data set.

Although the maximal curation strategy does reduce the number of larger data sets available to work with, there are still 34 IC_50_ data sets and 26 *K*_*i*_ data sets containing at least 500 compounds. These are composed of data from at least 14 (IC_50_) or 16 (*K*_*i*_) assays. As seen in the previous sections, these data sets definitely still contain some noise, but they are considerably less likely to contain wildly inconsistent results than those produced by more minimal curation schemes and are better suited to serve as a basis for further analysis or building and validating ML approaches.

## Conclusions

We have shown that combining literature data from different assays that measure IC_50_ values against what is nominally the same target can result in very large amounts of noise. More careful automated curation of the data sets using metadata available in ChEMBL (maximal curation scheme) can substantially reduce the overall noise level in combined data sets with either IC_50_ or *K*_*i*_ as the readout, at the expense of including substantially fewer data points. It is worth pointing out that even with the maximal curation settings, a significant amount of noise remains in the combined data sets.

While doing this work, we were surprised by the lack of consistency in the *K*_*i*_ data sets. We came to the project with the expectation to observe more interassay variability in the IC_50_ data than in the *K*_*i*_ data. However, the results did not meet this expectation (particularly before we manually pruned a large set of the data due to issues with the primary data source). It seems that although there are scientific reasons (such as different substrate concentrations) that render the combination of IC_50_ assays problematic, these are perhaps overwhelmed by practical problems when working with large collections of data drawn from patents and publications.

Good scientific practice requires some level of curation when combining data from different assays into a single data set for analysis (or training of ML models). We have demonstrated here that simplistic exports of data from resources such as ChEMBL can result in data sets that combine assays measured against different variants of the same protein or under different conditions. Without the necessary curation, we are left analyzing or building ML models on data sets that, in the best case, contain overwhelming amounts of noise. In the worst case, they do not make scientific sense. Although some level of irreducible noise remains given the experimental variability, the inevitable variation between laboratories, errors in the scientific literature, and the limits of what is possible when data sets are manually curated from the literature, we consider the maximal curation settings an important step forward toward high-quality public bioactivity data sets for training or validating ML models.

## Data Availability

The Jupyter notebooks used for this analysis, as well as the IC_50_ and *K*_*i*_ data sets discussed in the ”Impact of Curation on Data Set Size” section are available under an open-source license in our public GitHub repository: https://github.com/rinikerlab/overlapping_assays.

## Supporting Information Available

Impact of the individual curation steps on data set size, list of the ChEMBL32 assays that were excluded when constructing the ”pruned” *K*_*i*_ data sets, and additional results for minimal curation of the *K*_*i*_ assays as well as for the IC_50_ and *K*_*i*_ data sets when assays with up to 1000 compounds are included (PDF)
