# Supplementary material for: Combining IC50 or Ki Values from Different Sources Is a Source of Significant Noise
Source: J Chem Inf Model. 2024 Feb 23;64(5):1560–7. doi: 10.1021/acs.jcim.4c00049 (PMC10934815; doi:10.1021/acs.jcim.4c00049)
Supplement: Supplementary file 1 — ci4c00049_si_001.pdf [file ci4c00049_si_001.pdf]

SUPPORTING INFORMATION
Combining IC_{50} or K_i Values From Different Sources is a Source of Significant Noise

Gregory A. Landrum,* and Sereina Riniker*

Department of Chemistry and Applied Biosciences, ETH Zürich, Vladimir-Prelog-Weg 2, 8093 Zürich, Switzerland. E-mail: glandrum@ethz.ch, sriniker@ethz.ch

Table S1: Impact of the individual curation steps on data set size. The curation steps themselves are described in the Methods section in the main text.

Curation step	Number of assay pairs	Number of compound pairs
Assay size	1776	49047
Activity curation	1358	38022
Duplicate docs	642	13078
Remove mutants	1582	43656
Assay type	1583	43730
Assay metadata	698	19746
Only documents	1593	42951
Remove max assay size	2172	71077

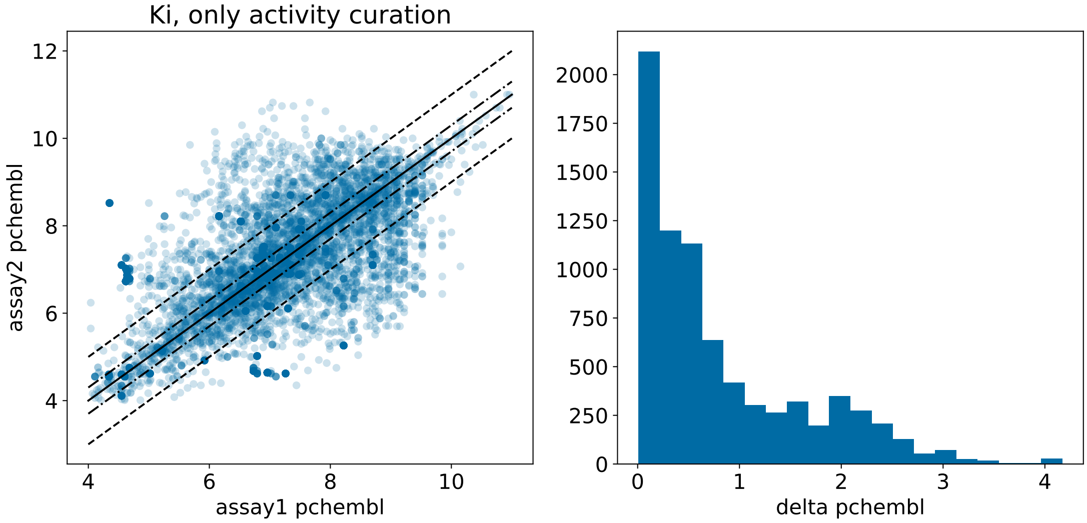

Figure S1: Agreement between duplicate measurements in K_i assays on the same target with only activity curation. The left pane shows the correlation plot between pchembl values from the two assays while the right pane has a histogram of Δpchembl, the differences in pchembl values. The solid black line in the left pane corresponds to $x = y$, the dot-dash lines mark a difference of 0.3, and the dashed line marks a difference of 1.0.

0.1 Assays Not Considered in the "Pruned" Set

This is the collection of 239 K_i assays which were not included in the "pruned" sets: CHEMBL3101211, CHEMBL2445070, CHEMBL2327206, CHEMBL2327942, CHEMBL3407119, CHEMBL3407118, CHEMBL3407117, CHEMBL3407116, CHEMBL3367569, CHEMBL3367568, CHEMBL3367567, CHEMBL3367566, CHEMBL3367565, CHEMBL3270420, CHEMBL3270419, CHEMBL3270418, CHEMBL3270416, CHEMBL3270415, CHEMBL3102349, CHEMBL3102348, CHEMBL3101213, CHEMBL3101212, CHEMBL3101210, CHEMBL3789760, CHEMBL3782912, CHEMBL3782911, CHEMBL3782910, CHEMBL3782909, CHEMBL3767552, CHEMBL3767551, CHEMBL3767550, CHEMBL3767549, CHEMBL3767548, CHEMBL3761242, CHEMBL3761241, CHEMBL3761240, CHEMBL3760464, CHEMBL2327205, CHEMBL3404500, CHEMBL3404499, CHEMBL3404498, CHEMBL3407120, CHEMBL4035751, CHEMBL4035750, CHEMBL4035749, CHEMBL4035748, CHEMBL4427979, CHEMBL4427978, CHEMBL3607055, CHEMBL3607054, CHEMBL3607053, CHEMBL3607052, CHEMBL3425083, CHEMBL3395808, CHEMBL4427980, CHEMBL3395806, CHEMBL3395807, CHEMBL4001628, CHEMBL4001627, CHEMBL4001626, CHEMBL4001625, CHEMBL3749744, CHEMBL3749743, CHEMBL3748431, CHEMBL3748430, CHEMBL2185518, CHEMBL1645695, CHEMBL1645694, CHEMBL993110, CHEMBL1015321, CHEMBL1015322, CHEMBL1015327, CHEMBL1063125, CHEMBL1063126, CHEMBL1063127, CHEMBL1063128, CHEMBL1038577, CHEMBL1038578, CHEMBL1038579, CHEMBL1063842, CHEMBL1063843, CHEMBL1947439, CHEMBL1815994, CHEMBL1815993, CHEMBL1815988, CHEMBL1815987, CHEMBL1645698, CHEMBL1645697, CHEMBL1645696, CHEMBL1002983, CHEMBL1002984, CHEMBL1002985, CHEMBL1002986, CHEMBL993108, CHEMBL993109, CHEMBL1947440, CHEMBL1947441, CHEMBL2049250, CHEMBL2049249, CHEMBL1031357, CHEMBL898699, CHEMBL2379886, CHEMBL1031353, CHEMBL1031354, CHEMBL1031355,

CHEMBL2049253, CHEMBL1031356, CHEMBL2049251, CHEMBL1678663, CHEMBL1064389, CHEMBL1678662, CHEMBL1678666, CHEMBL1678667, CHEMBL2380058, CHEMBL2380059, CHEMBL2380060, CHEMBL2380061, CHEMBL865638, CHEMBL895192, CHEMBL895191, CHEMBL862664, CHEMBL863824, CHEMBL863823, CHEMBL861561, CHEMBL861560, CHEMBL861559, CHEMBL864357, CHEMBL896308, CHEMBL896309, CHEMBL911283, CHEMBL862670, CHEMBL911284, CHEMBL911285, CHEMBL862669, CHEMBL911286, CHEMBL862671, CHEMBL829846, CHEMBL827186, CHEMBL829640, CHEMBL829822, CHEMBL828815, CHEMBL658750, CHEMBL657039, CHEMBL828135, CHEMBL657967, CHEMBL657825, CHEMBL828932, CHEMBL830339, CHEMBL3789758, CHEMBL828071, CHEMBL657150, CHEMBL3789752, CHEMBL3789754, CHEMBL3789755, CHEMBL828925, CHEMBL4513082, CHEMBL4495582, CHEMBL876689, CHEMBL832583, CHEMBL838574, CHEMBL839013, CHEMBL839012, CHEMBL658217, CHEMBL4651403, CHEMBL4731750, CHEMBL840160, CHEMBL827205, CHEMBL827204, CHEMBL830313, CHEMBL830312, CHEMBL876374, CHEMBL658042, CHEMBL657828, CHEMBL657153, CHEMBL662724, CHEMBL657821, CHEMBL1108468, CHEMBL657146, CHEMBL658749, CHEMBL867755, CHEMBL870709, CHEMBL657028, CHEMBL824221, CHEMBL3737169, CHEMBL873200, CHEMBL824219, CHEMBL824220, CHEMBL657976, CHEMBL3737173, CHEMBL657848, CHEMBL3737172, CHEMBL3737170, CHEMBL870708, CHEMBL657970, CHEMBL657149, CHEMBL657025, CHEMBL4303805, CHEMBL827937, CHEMBL657824, CHEMBL4303810, CHEMBL653170, CHEMBL658942, CHEMBL828069, CHEMBL875708, CHEMBL839950, CHEMBL875538, CHEMBL1217310, CHEMBL657042, CHEMBL1960463, CHEMBL1960462, CHEMBL1960461, CHEMBL659048, CHEMBL657145, CHEMBL1217308, CHEMBL1217309, CHEMBL1106525, CHEMBL2415808, CHEMBL2429043, CHEMBL4303819, CHEMBL3116664, CHEMBL3116665, CHEMBL2429044, CHEMBL3116666, CHEMBL828074, CHEMBL2429045, CHEMBL2429046, CHEMBL2429047, CHEMBL3116663, CHEMBL2429048, CHEMBL3116661, CHEMBL657820, CHEMBL2415807, CHEMBL827065, CHEMBL2415806, CHEMBL3116662, CHEMBL828905, CHEMBL828908, CHEMBL3562119, CHEMBL3561990

0.2 Analysis Using Assays With up to 1000 Compounds

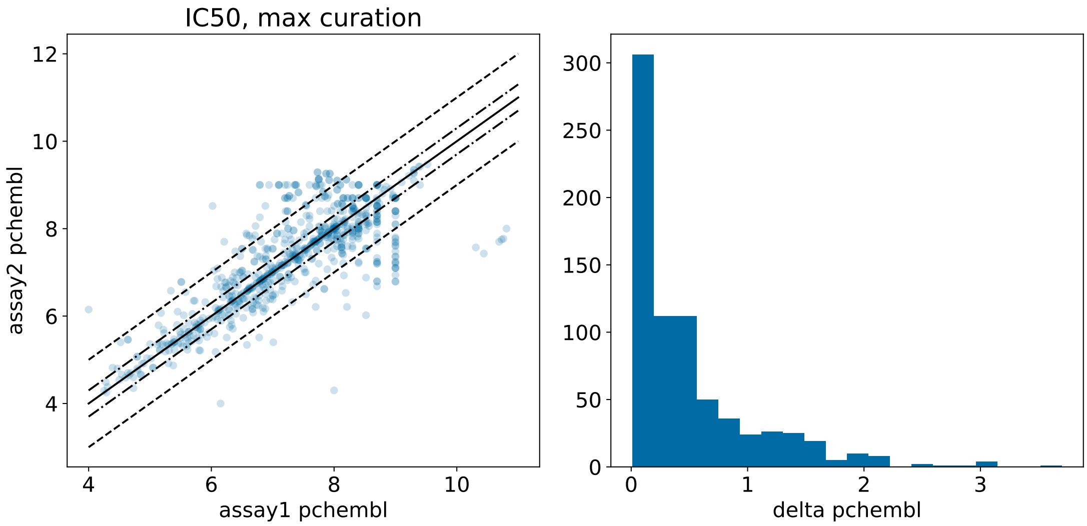

Figure S2: Agreement between duplicate measurements in IC_{50} assays on the same target with maximal curation. These plots include assays with up to 1000 compounds. (Left): Correlation plot between *pchembl* values from the two assays. The solid black line corresponds to $x = y$, the dot-dashed lines mark a difference of 0.3, and the dashed line marks a difference of 1.0. The regions outlined with red boxes are discussed in the text. (Right): Histogram of Δpchembl, the differences in *pchembl* values.

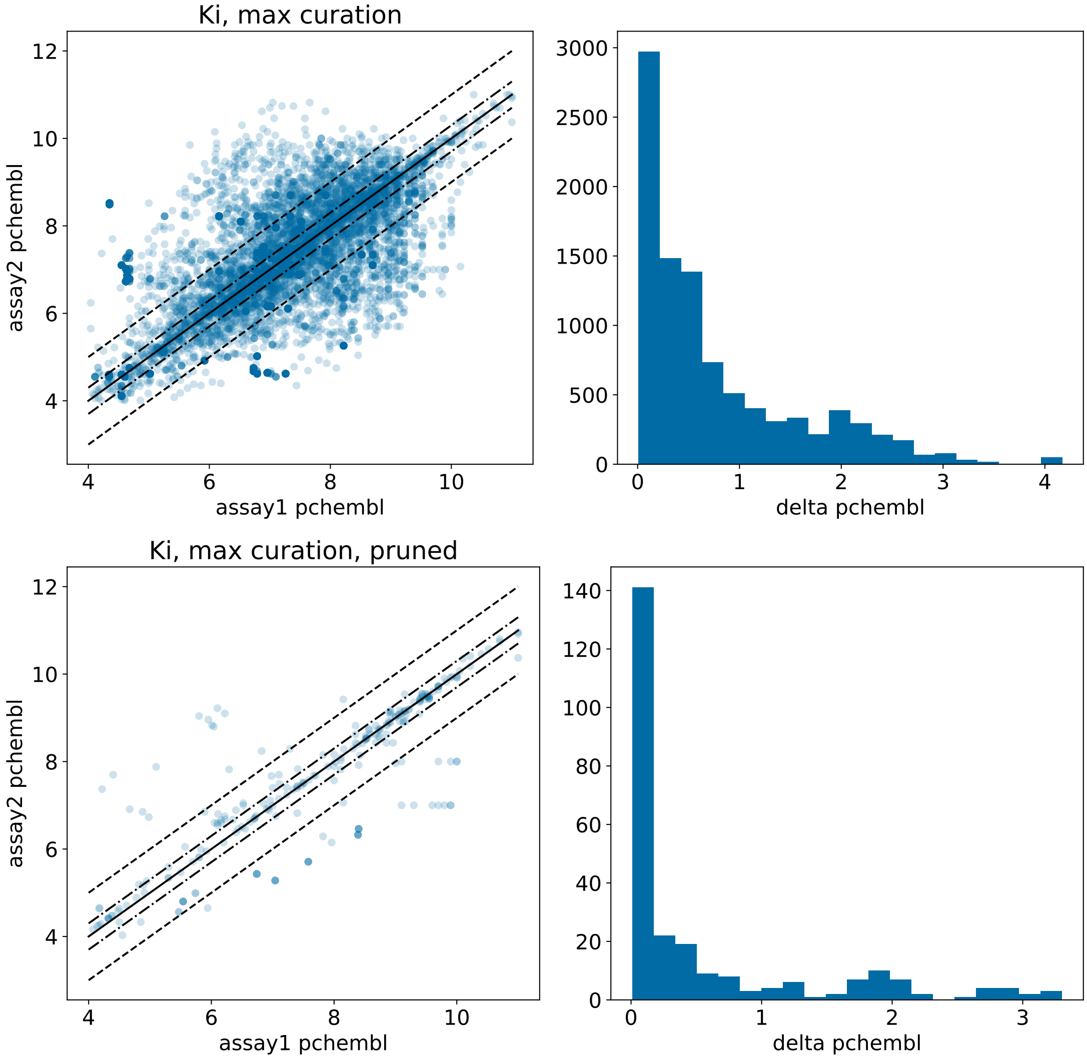

Figure S3: Agreement between duplicate measurements in K_i assays on the same target with maximal curation (top) and with 239 problematic assays (see text) removed (bottom). These plots include assays with up to 1000 compounds. (Left): Correlation plot between *pchembl* values from the two assays. The solid black line corresponds to $x = y$, the dot-dashed lines mark a difference of 0.3, and the dashed line marks a difference of 1.0. The regions outlined with red boxes are discussed in the text. (Right): Histogram of Δpchembl, the differences in *pchembl* values.
